# Interorgan Communications in Skeletal Pathophysiology: From Molecular Pathways to Multidisciplinary Therapies

**DOI:** 10.34133/research.1319

**Published:** 2026-06-24

**Authors:** Donghao Gan, Lei Qin, Guozhi Xiao

**Affiliations:** ^1^ Department of Biochemistry, Homeostatic Medicine Institute, School of Medicine, Guangdong Provincial Key Laboratory of Cell Microenvironment and Disease Research, Shenzhen Key Laboratory of Cell Microenvironment, Southern University of Science and Technology, Shenzhen, China.; ^2^ Department of Orthopaedics and Rehabilitation, Yale School of Medicine, New Haven, CT, USA.; ^3^ Department of Orthopaedics, Shenzhen Nanshan People’s Hospital, Affiliated Nanshan Hospital of Shenzhen University, Shenzhen, China.

## Abstract

Communications between organs contribute to propel physiological functions, as well as pathological processes. Growing evidence indicates that bone is continuously regulated by multiple layers of endocrine, immune, metabolic, and neural networks. The common bone diseases, including osteoporosis, osteoarthritis, rheumatoid arthritis, and intervertebral disc degeneration, are reported with tight associations to other nonbone organs. These organ–bone axes emphasize the interorgan communications that maintain the bone homeostasis and maintain individual health. In this review, we systematically summarize recent advances in the organ–bone communications, highlighting mechanistic innovations, such as the gut–bone–immune axis, neuro–immune–bone integration, hypothalamic–pituitary–adrenal axis, adipo–neuro–bone axis, and their connections with bone pathophysiology. Furthermore, we highlight circulating biomarkers and imaging advances reflecting organ–bone crosstalk. Finally, we discuss the clinical relevance, current research gaps, and unsolved questions of organ–bone communications in the diagnostic stratification, treatment selection, and multidisciplinary management of bone diseases.

## Introduction

Bone diseases are a major component of the global public health burden. Represented by osteoporosis (OP), osteoarthritis (OA), rheumatoid arthritis (RA), and intervertebral disc degeneration (IVDD), their prevalence is rising in tandem with population aging, obesity, and changing lifestyles. OP and fragility fractures lead to a high risk of disability, refracture, and mortality and incur substantial direct and indirect costs. It is estimated that the total number of OP cases worldwide will reach up to 263.2 million between 2030 and 2034, placing substantial pressure on healthcare systems [1]. Moreover, according to the latest Global Burden of Disease Study (GBD 2021), the number of people living with OA had more than doubled between 1990 and 2020 and is projected to reach about 1 billion by 2050 [2]. OA has become one of the major chronic diseases leading to increased years lived with disability, resulting in billions of dollars in medical expenses and unemployment losses each year. Furthermore, RA affects approximately 17.6 million people worldwide with influences in joints and multiple systemic conditions, substantially increasing the risk of cardiovascular complications and premature death [3]. In addition, IVDD links to low back pain, which affected 619 million people in 2020, with a projection of 843 million by 2050 [4]. These rising trends and incidence of bone diseases highlight the need for improved prevention and treatment strategies.

Bone is not an independent organ and bone health is not a “local issue”. Bone is a highly metabolic and dynamic tissue, which is constantly regulated by multiple layers of endocrine, immune, metabolic, and neural networks. Historically, bone metabolism was first understood from an endocrine and mechanical perspective. Subsequently, the concept of the organ–bone axis gradually became clear: liver–bone (vitamin D 25-hydroxylation, insulin-like growth factor 1 [IGF-1]) [5], kidney–bone (calcium and phosphorus homeostasis, fibroblast growth factor 23 [FGF23]/parathyroid hormone [PTH]) [6], adipose–bone (leptin, adiponectin, and bone marrow adipose) [7], gut–bone (microbiome and mucosal immunity) [8,9], endocrine–bone (pancreas, thyroid, and adrenal glands) [10], cardiovascular–bone (angiogenesis and endothelial function) [11], neural–bone (central nervous system-sympathetic / parasympathetic-sensory nerves-neurotrophic factors) [12], and muscle–bone (mechanotransduction and myokines/motor factors) [13]. These axes collectively maintain bone homeostasis and systemic health.

Circulating factors from bone, kidney, liver, muscle, adipose tissue, gut, and the immune system now serve as windows into organ–bone crosstalk (Table 1). Importantly, these communications are fundamentally bidirectional: Bone not only responds to systemic signals but also actively regulates remote organ physiology through endocrine factors termed osteokines. These dual roles underscore bone as both a target and a driver of multiorgan homeostasis and diseases.

**Table 1.** Circulating and imaging biomarkers reflecting organ–bone crosstalk

Organ/Source	Biomarker	Key functions	Clinical relevance	References
Osteocytes	Sclerostin (total/bioactive)	Inhibits Wnt signaling in bone formation; linked to fracture risk and cardiovascular traits via genetic/observational studies; target of romosozumab.	Fracture prediction; interpret with P1NP/CTX for enhanced value; standardization ongoing.	[14,169]
Kidney–bone axis	Fibroblast growth factor 23 (FGF23)	Regulates phosphate homeostasis; elevates early in CKD-MBD before phosphate/PTH changes; associates with cardiac remodeling and mortality.	Early CKD-MBD detection; prognostic for adverse outcomes in renal disease.	[18]
	Lipocalin 2	Bone-derived; modulates appetite and insulin sensitivity; reflects bidirectional kidney–bone signaling.	Insights into metabolic-renal–bone loops; emerging in diabetes/kidney disorders.	[170]
Adipose tissue	Leptin (adipokine)	Supports osteoblast activity at physiologic levels; obesity-associated hyperleptinemia suppresses bone formation via central sympathetic pathways.	Correlates with obesity-related bone loss; potential for metabolic syndrome monitoring.	[171]
Endocrine–bone axis	Osteocalcin	Bone-derived hormone; regulates glucose metabolism and links bone to pancreas.	Marker of bone-endocrine crosstalk; associations with diabetes and fertility.	[15,172]
Liver	Bone morphogenetic protein 9 (BMP9)	Circulating factor; involved in liver–bone signaling; biomarker for nonalcoholic fatty liver disease (NAFLD) stratification.	Noninvasive NAFLD assessment; potential links to hepatic osteodystrophy.	[16]
Gut (microbiome)	Short-chain fatty acids (SCFAs), bile acids	Microbial metabolites; influence immune-mediated bone remodeling; microbiome shifts correlate with bone turnover.	Probiotic interventions (e.g., *Limosilactobacillus reuteri*) may attenuate bone loss in select cohorts, though heterogeneous.	[173,174]
Muscle	Irisin	Myokine; promotes bone formation; lower levels linked to osteoporosis/fracture in women; associates with fall risk and microarchitecture.	Investigational biomarker for sarcopenia–osteoporosis and frailty-related bone loss.	[175]
Immune system	Cytokines (TNF, IL-17); RANKL/OPG axis	Drive inflammatory bone resorption; measurable in circulation; therapeutically targetable (e.g., anti-TNF agents).	Risk stratification in autoimmune diseases; pairs with bone markers for trial enrichment.	[176]

CKD-MBD, chronic kidney disease–mineral and bone disorder; TNF, tumor necrosis factor; IL-17, interleukin-17; PTH, parathyroid hormone; RANKL, receptor activator of nuclear factor κB ligand; OPG, osteoprotegerin; P1NP, procollagen type I N-terminal propeptide; CTX, C-terminal telopeptide of type I collagen

On one hand, bone-derived molecules are emerging as clinically relevant biomarkers for metabolic, cardiovascular, liver, and renal disorders. Sclerostin, secreted predominantly by osteocytes and the therapeutic target of romosozumab, is associated with fracture risk and cardiovascular phenotypes across genetic, observational, and interventional studies [14]. Osteocalcin modulates glucose metabolism and links bone to endocrine organs [15]. Circulating bone morphogenetic protein 9 (BMP9) has been proposed as a biomarker for noninvasive stratification of nonalcoholic fatty liver disease [16]. Moreover, our lab demonstrates that Pinch-dependent Cxcl12 production by bone marrow stromal cells regulates hepatic Mbl2 expression, thereby coordinating hematopoietic development and innate immune defense, with disruption of this pathway leading to profound immune failure and lethality [17]. Furthermore, FGF23 primarily secreted by osteocytes, rises early in chronic kidney disease–mineral and bone disorder (CKD-MBD), often before phosphate or PTH change, and tracks adverse cardiac and survival outcomes [18].

On the other hand, other factors derived from nonbone tissues and organs also link the healthy and disturbed conditions in our body. Adipokines, like leptin, from adipose tissue promote osteoblast activity, but excess adipokines correlate with obesity-induced bone resorption [19]. Moreover, gut-derived short-chain fatty acids (SCFAs) influence immune-mediated bone remodeling [20]. Immune-derived mediators, particularly tumor necrosis factor (TNF), interleukin-17 (IL-17), and the receptor activator of nuclear factor κB ligand (RANKL)/osteoprotegerin (OPG) axis, leave a measurable molecular imprint on bone; in inflammatory diseases, pairing cytokine profiles with bone turnover markers improves risk stratification and clinical trial enrichment [21]. These biomarkers enable early detection of systemic imbalances.

The interorgan axes between bone and other soft organs work together to maintain skeletal homeostasis and influence the patterns of orthopedic diseases. Collectively, these findings indicate that bone diseases should no longer be viewed as isolated skeletal disorders but rather as manifestations of systemic interorgan network dysregulation. In this review, we systematically summarize recent advances in the influence of major organs on the development and progression of bone diseases, highlighting emerging mechanistic insights into multiorgan communication networks—such as the gut–bone–immune axis, neuro–immune–bone axis, hypothalamic–pituitary–adrenal (HPA) axis, and adipo–neuro–bone axis—and their pathophysiological integration in OP, OA, RA, and IVDD. Furthermore, we discuss the clinical importance of organ–bone axes in diagnostic stratification, therapeutic targeting, and translational potential.

## Principles of Organ–Bone Crosstalk Mediators

**Fig. 1.**
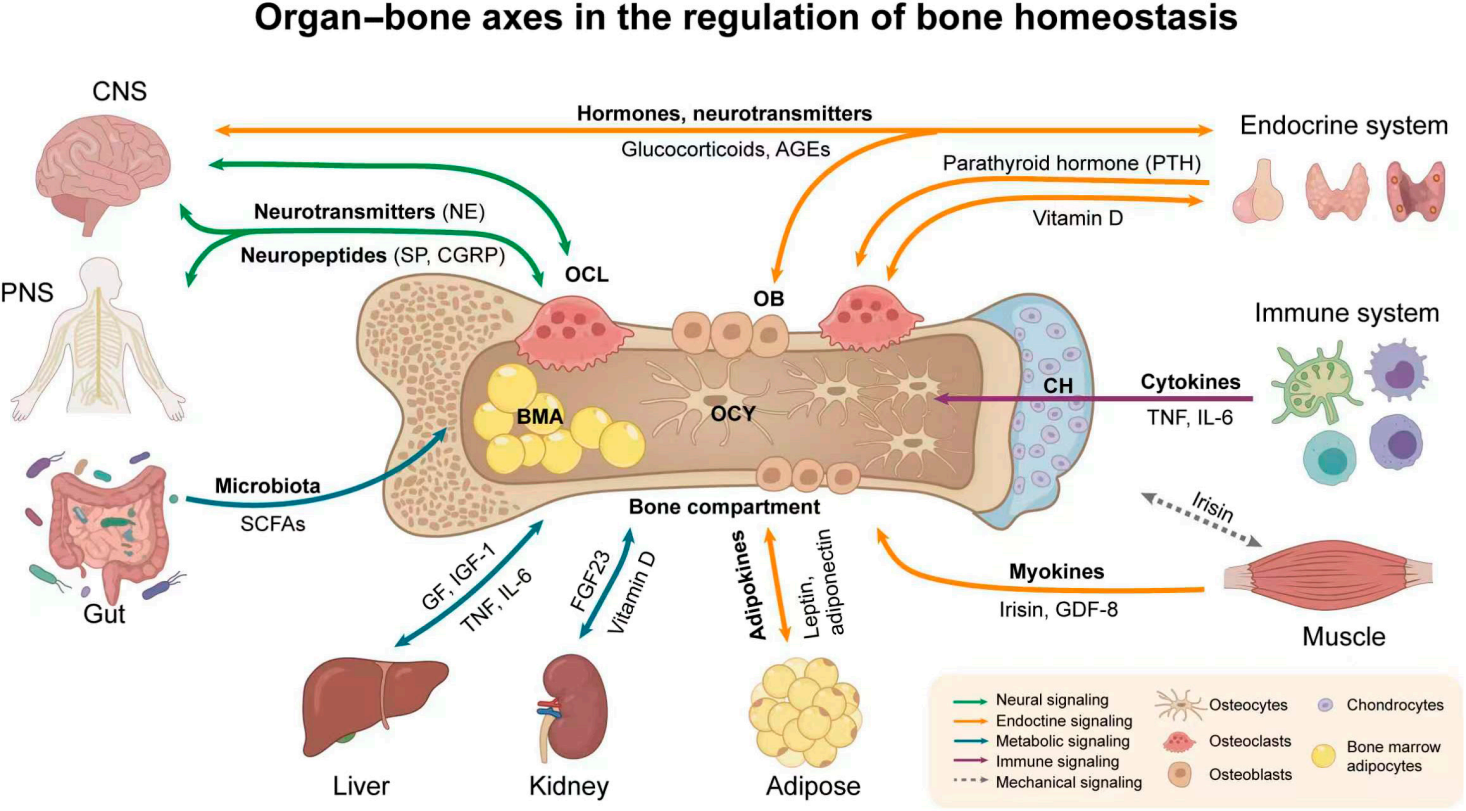
Overview of organ–bone axes in the regulation of bone homeostasis. Multiple organs (including neural system, endocrine organ, liver, kidney, adipose tissue, gut, immune system, and muscle system) interact with bone with direct and indirect communications over bone cells (including chondrocytes, osteoclasts, osteoblasts, and osteocytes) during skeletal pathophysiology.

Bones provide the structural framework for the body and enable movement, but they are also highly dynamic organs intricately connected to systemic homeostasis. Increasing evidence shows that skeletal disorders are not only the result of intrinsic bone defects but also arise from disturbances in other organs or systems (Fig. 1). The liver, kidney, adipose tissue, cardiovascular system, nervous system, and muscles all exert profound influences on bone. Rather than acting in isolation, these organs communicate with the skeleton through shared pathways (Table 1). This section establishes the fundamental principles of organ–bone crosstalk by categorizing the primary mediators—endocrine, neural, immune, and mechanical—that shape bone integrity.

### Endocrine and metabolic mediators (liver, kidney, adipose, gastrointestinal, and endocrine)

The skeleton is heavily regulated by a network of systemic hormones and metabolic factors derived from distant organs. The liver and kidneys function as the primary metabolic hubs for bone mineral balance. The liver carries out the first step of vitamin D activation through 25-hydroxylation [22] and produces IGF-1 under the control of growth hormones to support bone growth and strength [23].

Beyond classical mineral metabolism, adipose tissue and other endocrine organs release potent metabolic signals. Adipose tissue secretes adipokines, such as leptin and adiponectin. Leptin regulates bone metabolism by suppressing bone formation and enhancing resorption via central sympathetic activation [24], while adiponectin generally exerts complex (often inhibitory) actions on bone mass [25]. Importantly, we demonstrate that sclerostin derived from adiponectin-expressing adipose lineage cells negatively regulates bone mass during adulthood, and its selective deletion increases trabecular bone accrual without affecting adiposity or glucose metabolism [26]. We also showed that adipocyte-expressed Kindlin-2 promotes age- and diet-associated bone loss by activating the fatty acid synthase–peroxisome proliferator-activated receptor γ–fatty acid-binding protein 4 pathway and suppressing insulin signaling, whereas adipose-specific Kindlin-2 inhibition enhances bone mass [27,28]. Additionally, bone marrow adipose tissue acts as a metabolically active depot associated with bone mass [29].

Classic endocrine glands also dictate bone turnover. For instance, the thyroid gland controls bone balance through thyroid hormones and BMP signaling [10,30]. Further study showed that excess exogenous glucocorticoids inhibit murine bone turnover by impairing fatty acid transportation in the skeletal microenvironment [31].

### Neural mediators (central nervous system and peripheral nervous system)

The nervous system finely tunes bone health through both local peripheral innervation and central global regulation. The peripheral nervous system supplies bone with sensory, sympathetic, and parasympathetic fibers. Sensory nerves (Aδ and C fibers) release neuropeptides like substance P (SP) and calcitonin gene-related peptide (CGRP), which shape bone remodeling, stimulate osteoblast differentiation, and are vital for fracture healing [32]. This intricate interplay between sensory nerves and bone tissue cells is of paramount importance in the field of bone biology and pathology. Mechanistically, SP can enhance osteoclastogenesis and stimulate late-stage osteoblast activity and inflammatory signaling, so its net effect depends on dose and disease state [33]. SP also contributes to cartilage and bone pathology in arthritis [34]. Furthermore, sensory nerves are closely coupled to angiogenesis in bone, promoting tissue repair and regeneration, whereas aberrant neurovascular expansion can contribute to pathological remodeling and disease progression [35].

Conversely, sympathetic nerves release norepinephrine, activating β2-adrenergic receptors on osteoblasts and osteoclasts to generally inhibit bone formation and promote resorption [36]. Providing a physiological counterbalance, parasympathetic (cholinergic) innervation utilizes acetylcholine through muscarinic receptors (M3/M5) to enhance osteoblast differentiation and limit osteoclast activity [37,38]. During bone formation, cholinergic sympathetic fibers contact osteocytes and osteoprogenitors, preserve osteocyte survival, and contribute to exercise-induced bone accrual [39].

At the macro level, the central nervous system oversees bone regulation primarily through the hypothalamus, integrating signals from the body to adjust skeletal metabolism. The hypothalamus integrates systemic signals and controls the HPA axis, linking systemic stress responses directly to bone mass regulation. Stress activates it, releasing cortisol via the hypothalamus, pituitary, and adrenals [40]. High cortisol promotes bone loss by increasing resorption and cutting formation, raising OP risk [41]. Direct nerve–bone contacts further supply neurotrophins, such as nerve growth factor (NGF) and brain-derived neurotrophic factor, which regulate local neuronal survival and actively participate in stem cell differentiation and bone regeneration [42,43]. Recent discoveries of skeletal interoceptive circuits have further emphasized the critical role of the central nervous system in the regulation of bone homeostasis. Specifically, osteoblast-derived prostaglandin E2 (PGE2) activates EP4 receptors on sensory nerves to relay bone mechanical status to the hypothalamus, where it suppresses sympathetic output and promotes bone formation [44].

### Immune and inflammatory mediators

Systemic and local immune environments are inextricably linked to skeletal health. Immune and inflammatory mediators, often transported via the cardiovascular system, dictate the balance of osteoclastogenesis. The bone vasculature is central to coordinating bone formation and repair, with endothelial cells promoting healing through vascular endothelial growth factor/Notch signaling [45]. Distant organs are major sources of these inflammatory mediators. In the liver, activated Kupffer cells and injured hepatocytes can release proinflammatory cytokines, including TNF-α and IL-6, which secondarily amplify RANKL-driven osteoclastogenesis [5]. Similarly, expanded adipose tissue in obese states releases leptin, IL-6, and TNF, which drive systemic low-grade inflammation that compromises joint and subchondral bone integrity [46].

### Mechanical signals (muscle)

The muscular system is the primary source of mechanical loading, a fundamental requirement for skeletal homeostasis in accordance with Wolff’s law. Muscle-derived mechanical loading activates mechanosensors in osteocytes and osteoblast-lineage cells, including integrins and Piezo1, thereby inducing Ca^2+^-dependent signaling and Wnt/β-catenin-associated osteogenic pathways that promote osteoblast activity [47,48]. Loss of these mechanical signals, such as during immobilization or microgravity, rapidly tips the balance toward bone resorption. Prolonged bed rest, sarcopenia-associated inactivity, and immobilization after injury reduce mechanical loading on the skeleton, impair bone formation, and shift bone remodeling toward resorption [49]. Moreover, resistance exercise boosts bone formation by raising strain levels above normal [50].

Beyond mechanical loading, muscles communicate with bone through the release of myokines. Exercise-responsive myokines participate in muscle–bone crosstalk and regulate exercise-associated bone remodeling, whereas chronic inflammatory signaling in wasting or inflammatory states may shift bone remodeling toward impaired bone formation and enhanced resorption [51]. Among these factors, irisin promotes osteoblast differentiation and increases cortical bone mass [52], while FGF2 supports osteogenesis and angiogenesis [13,53]. In contrast, myostatin acts as a negative regulator of musculoskeletal homeostasis, and its inhibition improves bone mass [54]. Together, these mechanical and biochemical signals underscore the necessity of muscle–bone crosstalk in maintaining skeletal integrity.

## Multiorgan Axes in Skeletal Homeostasis

Bone pathology arises from both internal defects and intricate interactions with distant organs. The liver, kidney, endocrine glands, neural circuits, immune system, adipose tissue, muscle, and gut microbiota each provide distinct but interconnected regulatory signals. These inputs shape bone remodeling through hormones, cytokines, and metabolites. Here, we summarize current updated multiple-organ axes in bone and bone-related diseases, highlighting the interorgan communications.

### Gut–bone–immune axis

The gut microbiota is now recognized as a bidirectional interface interacting with the gastrointestinal, immune, endocrine, and nervous systems, thereby influencing cellular behavior across multiple organs. Through metabolites, immune modulation, and endocrine/neuroendocrine signaling, the gut microbial community exerts cross-organ control over the rhythmicity of bone remodeling—a concept collectively referred to as the gut–bone–immune axis. Recent systematic reviews and multicohort, multiomics datasets show that microbial dysbiosis not only perturbs the balance between bone formation and resorption but also resets immune thresholds within niches such as bone marrow and synovium. These shifts give rise to “immuno-metabolic–endocrine” phenotypes that underlie OP, inflammatory bone loss, and degenerative joint disease [55].

Metabolite signaling stands at the center of this axis, for example SCFAs. Converging evidence from murine models and human intervention studies supports a unified axis in which high-fiber intake increases SCFAs, lowers osteoclast activity, and ultimately improves bone mass [56]. Bile acids (BAs), synthesized in the liver and further modified by intestinal microbes, form an important regulatory tier in skeletal homeostasis. Microbial remodeling of the BA pool activates bone-associated FXR and TGR5 pathways, leading to suppression of osteoclastogenesis and enhancement of osteoblast activity and angiogenic–osteogenic coupling. Studies in estrogen-deficiency and bone-repair models suggest that targeting BA signaling or reshaping the gut microbiota may offer therapeutic benefit in conditions marked by pathological bone loss [20]. Tryptophan metabolism provides another major route by which the gut microbiota communicates with the skeleton. Microbial conversion of dietary tryptophan generates indoles, kynurenines, and tryptamine, each capable of shaping mucosal and systemic immunity [57]. Indole derivatives engage the aryl hydrocarbon receptor (AhR), enabling intestinal signals to alter macrophage and dendritic-cell behavior and to recalibrate the T helper 17 (Th17)/regulatory T cell (Treg) axis. These immune shifts converge on the RANKL/OPG pathway, thereby influencing osteoclast and osteoblast dynamics. Pharmacologic AhR inhibition has been shown to improve cortical bone quality in aging models, emphasizing its relevance to skeletal degeneration [58]. Disruption of innate immune sensing also plays a role: Mice lacking Toll-like receptor 9 develop dysbiosis and persistent low-grade inflammation, which enhances osteoclastogenesis and drives bone loss [59]. Multiple microbial taxa further sculpt the osteoimmune landscape. Th17-inducing organisms such as segmented filamentous bacteria enhance Th17-associated inflammatory signaling and may thereby facilitate osteoclastogenic bone resorption [60], whereas reductions in SCFA production weaken regulatory T cell-mediated bone-protective programs and shift the balance toward bone resorption [20]. Translational data in peri- and postmenopausal women support this concept: *Bacteroides vulgatus* is inversely associated with bone mineral density (BMD), while the SCFA-related metabolite valeric acid correlates positively with BMD. Experimental interventions confirm that *B. vulgatus* accelerates bone resorption and compromises bone microarchitecture, whereas valeric acid inhibits osteoclast maturation and enhances osteoblast differentiation [61]. Gut-derived hormones add yet another regulatory dimension. Incretins such as glucose-dependent insulinotropic polypeptide suppress osteoclastogenesis and improve osteoblast survival and function [62]. These findings have sparked interest in incretin-based analogs as potential therapeutics acting along a microbiota–gut hormone–bone axis to counter skeletal fragility.

### Neuro–immune–bone axis

The sensory neuro–immune axis operates as a critical bidirectional regulator in several important bone diseases, including OA and RA conditions. Sensory nerve endings lie in close apposition to synovial cells, and their reciprocal interactions shape not only the perception of pain but also the balance between inflammation and resolution. Viewing OA pain through a neuroimmunological lens has therefore provided a conceptual framework for “treating pain through immune modulation” [63]. Within the joint, sensory afferents release neuropeptides such as CGRP, the neurokinin-1 receptor ligand, and SP. Neurokinin-1 receptor signaling governs cartilage matrix turnover and sensitization to pain. CGRP and SP can directly reprogram synovial macrophages and chondrocytes, promoting proinflammatory transcriptional programs and up-regulating matrix-degrading enzymes [64,65]. Synovial macrophages and stromal cells, in turn, release cytokines and NGF that foster neurite sprouting and peripheral sensitization, establishing a self-reinforcing “pain–inflammation” feedback loop [66]. Both clinical and preclinical studies underscore the importance of CGRP and SP pathways in maintaining OA-associated cartilage dysregulation and pain perception. Therapeutic blockade of NGF signaling can reduce pain in osteoarthritis, although the long-term consequences for cartilage homeostasis require careful patient stratification [67]. A broader set of sensory ion channels—including voltage-gated sodium channels (Nav family) and transient receptor potential (TRP) channels—also contribute to inflammatory pain and neuroimmune coupling. Selective peripheral inhibition of these channels has shown promise in OA models, demonstrating not only analgesic potential but also improvement in structural joint integrity [68].

In the translational management of bone and joint diseases, the neuro–immune–bone axis has emerged as a multidimensional therapeutic target. One major direction is the precise modulation of neuropeptide signaling. NGF inhibitors and CGRP-pathway antagonists can provide substantial analgesic benefit; however, their use should be accompanied by imaging or liquid-biopsy–based monitoring of cartilage integrity and macrophage phenotypes to avoid a “pain relief–driven tissue injury” effect [35,69]. Another promising approach lies in integrating neuromodulation with immunotherapy. Techniques such as vagus nerve stimulation, sympathetic modulation, and localized neural blockade—when combined with disease-modifying antirheumatic drugs or anti-inflammatory agents—may collectively lower the threshold of neuroimmune coupling and improve disease control. Recalibrating circadian and endocrine rhythms also represents an actionable strategy. Together, these therapeutic concepts highlight a broader principle: Systemic regulation of the neuro–immune–bone axis provides a mechanistic bridge between basic biology and clinical intervention, opening a new framework for improving outcomes in skeletal and joint disorders.

### HPA axis

Chronic inflammation and persistent stress disrupt the physiological rhythm and feedback control of cortisol within the HPA axis [70], which is tightly coupled to circadian regulation. Circadian and sleep-related rhythms in turn govern daily variation in bone turnover markers [71,72] and modulate monocyte–macrophage and T-cell activity, thereby influencing inflammatory activity in RA and OA [73]. Chronic stress further compounds this burden by reducing BMD [74]. Chronotherapy with modified-release prednisone exploits this circadian framework by aligning glucocorticoid exposure with nocturnal inflammatory activity, improving morning stiffness and symptom control in RA [75].

Recent studies indicate that chronic psychological or physiological stress can exacerbate RA through neuro–immune–bone axis pathways [76]. Heightened sympathetic output during sustained stress increases catecholamine release, which in turn amplifies proinflammatory cytokine production. At the same time, impaired HPA-axis reactivity diminishes anti-inflammatory cortisol secretion and alters glucocorticoid receptor sensitivity, weakening the feedback loops between the hypothalamus and pituitary [77,78]. This combination fosters systemic immune imbalance and a lowered threshold for joint inflammation. HPA-axis dysfunction also shapes adaptive immunity. Altered glucocorticoid signaling can skew T-cell differentiation toward Th17 phenotypes, promoting their expansion and reinforcing downstream inflammatory cascades [79]. Together, these mechanisms position the HPA axis as a central regulator that links systemic stress responses with local joint pathology.

### Adipo–neuro–bone axis

Growing evidence indicates that bone homeostasis is not regulated solely by mechanical and endocrine signals but is tightly coordinated by a dynamic adipo–neuro–bone axis. Bone marrow adipocytes, peripheral adipose tissue, and sensory/autonomic nerve fibers form a highly interactive network that regulates osteoblast–osteoclast coupling, vascular remodeling, and force adaptation within the bone microenvironment. This axis exhibits bidirectional crosstalk: Adipokine secretion influences neuronal excitability and neuropeptide release, while neural signals, in turn, modulate lipid metabolism, adipocyte differentiation, and local inflammatory responses in bone.

Leptin serves as a typical example of adipose-derived regulators in adipo–neuro–bone axis. Leptin is produced by adipocytes, which can directly boost osteoblast and chondrocyte activity for bone growth, but it also indirectly regulates bone via the hypothalamus, sympathetic nervous system, body weight changes, and effects on other hormones like those from the pituitary gland [19]. Through the central nervous system, leptin inhibits bone formation using sympathetic pathways and serotonin modulation. Studies show that leptin deficiency lowers sympathetic tone, leading to higher bone mass, while β-adrenergic blockade or leptin replacement during disuse mitigates bone loss by boosting osteoblast activity, curbing osteoclast function, and maintaining leptin levels to preserve bone density and formation rates [80,81]. Additionally, leptin curbs serotonin synthesis in the brainstem, which otherwise promotes bone accrual through hypothalamic receptors. Disruption of this serotonin pathway corrects leptin-deficient bone phenotypes, which indicated a shared neural mechanism for bone mass, appetite, and energy balance [82].

OP is characterized by increased bone marrow adiposity, accompanied by reduced sensory innervation and impaired osteoblast activity, highlighting a dysfunction in the adipo–neuro–bone axis [32]. Under conditions, such as aging, estrogen deficiency, and chronic inflammatory states, excessive bone marrow fat accumulation creates a lipotoxic microenvironment enriched with saturated fatty acids and bioactive lipids [83]. These metabolites activate neuronal TRP channels, triggering neurogenic inflammation, which in turn promotes osteoclast recruitment and differentiation [84]. Together, these interactions form a self-reinforcing adipo–neuro–bone metabolic cycle.

Besides OP, adipo–neuro–bone axis also participates in other common bone diseases. In OA, periarticular adipose deposits, such as the infrapatellar fat pad, function as active neurometabolic hubs [85]. Adipokines, including leptin and visfatin, can induce the production of NGF, IL-6, and matrix-degrading enzymes in synovial fibroblasts and chondrocytes. This process enhances sensory nerve sensitivity and perpetuates a chronic pain-inflammation circuit [86]. In RA, systemic metabolic disturbances and inflamed synovial adipose tissue intensify the sympathetic–osteoclast coupling, thereby accelerating para-articular bone erosion [87]. Increased bone marrow adiposity further disrupts local neuro-osteogenic signaling, leading to persistent bone loss even under immunosuppressive therapy [88].

Therefore, leptin antagonists, adiponectin mimetics, and interventions that normalize the lipid composition of bone marrow adipose tissue may help rebalance neural excitability and skeletal metabolism. Selectively blocking sympathetic β-adrenergic signaling, inhibiting TRP channels activated by lipotoxic metabolites, or enhancing osteoanabolic signaling derived from sensory nerves could restore osteoblast–osteoclast coupling [89,90]. Combining antilipotoxic therapies (such as peroxisome proliferator-activated receptor γ modulators or ceramide pathway inhibitors) with anabolic agents (e.g., PTH analogs or sclerostin inhibitors) could disrupt the adipo–neuro–bone cycle and benefit bone health [91,92].

In summary, the adipo–neuro–bone axis provides a mechanistic framework linking metabolism, neural activity, and skeletal integrity. Understanding its components and signaling hierarchy not only enriches fundamental bone biology but also opens translational avenues for treating OP, OA, RA, and other metabolic bone disorders.

## Pathophysiological Integration in Skeletal Diseases

The multiorgan regulatory axes and their mediators do not act in isolation. When perturbed, these interconnected metabolic, immune, neuroendocrine, and mechanical pathways converge to shape the onset and progression of skeletal diseases. Disruptions along these axes manifest as distinct yet mechanistically overlapping clinical entities. This section illustrates how imbalances in metabolic, immune, neural, and mechanical crosstalk manifest in distinct clinical phenotypes, including osteoarthritis, RA, OP, and IVDD.

### OP

**Fig. 2.**
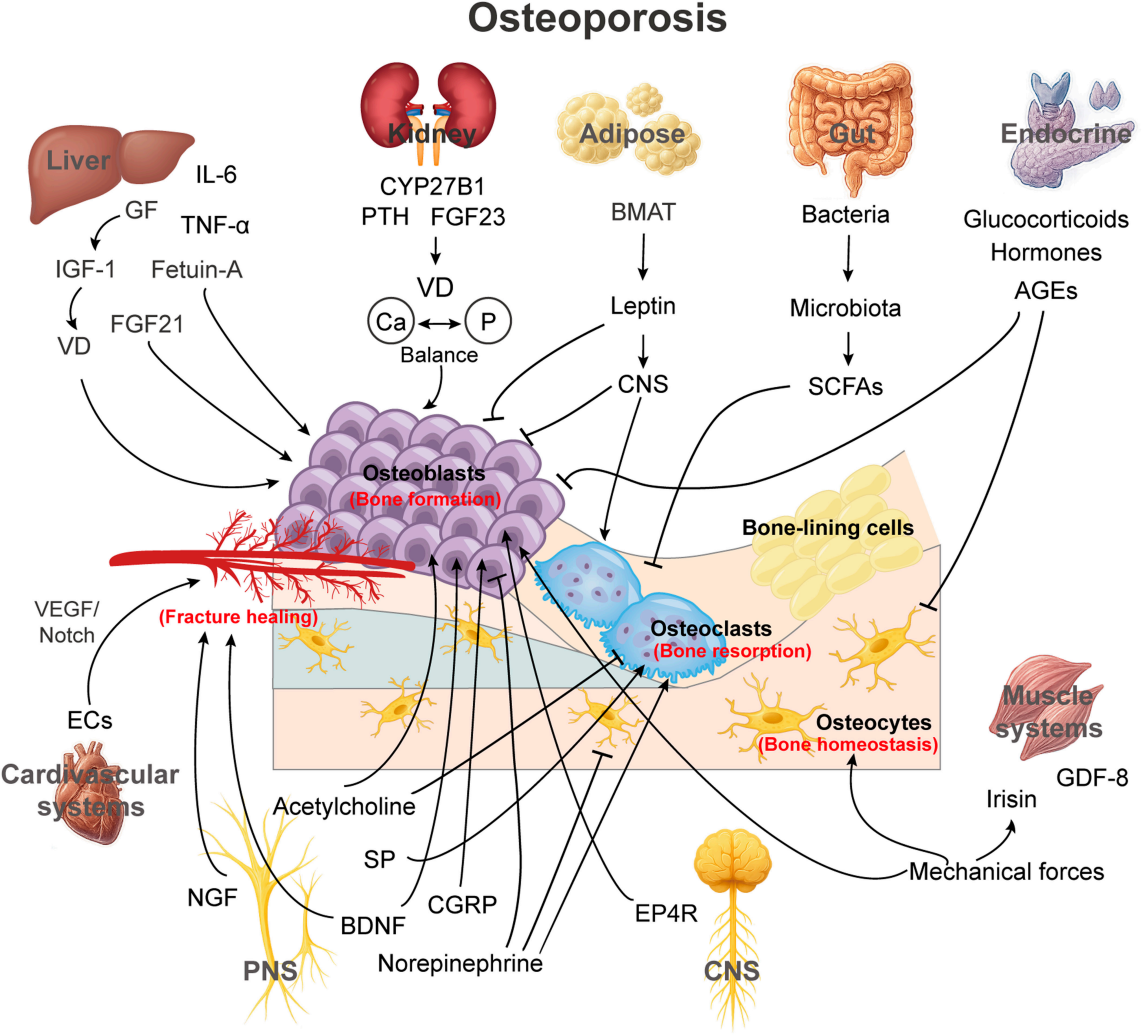
Organ–bone axes in regulating osteoporosis (OP) pathogenesis. Liver, kidney, and other organs contribute to bone remodeling through various secreted factors that directly or indirectly influence the formation and activities of osteoblasts, osteoclasts, bone-lining cells, osteocytes, and angiogenesis in bone environment. VEGF, vascular endothelial growth factor; BMAT, bone marrow adipose tissue; CNS, central nervous system; AGEs, advanced glycation end products.

OP is the classic manifestation of disrupted systemic endocrine and metabolic crosstalk (Fig. 2). For example, hepatic pathology contributes to bone loss across a disease spectrum: Nonalcoholic fatty liver disease is associated with modestly reduced BMD, while cirrhosis is accompanied by a substantially higher prevalence of OP. Mechanistically, hepatocyte-derived RANKL, up-regulated under inflammatory conditions, represents a direct liver-to-bone endocrine signal that promotes osteoclast-mediated resorption [93,94]. Concurrently, CKD promotes skeletal fragility through disturbances in mineral metabolism, bone turnover, and bone quality that evolve with declining kidney function [95]. The earliest identifiable alteration—often preceding changes in PTH, calcium, or active vitamin D—is a marked elevation in osteocyte-derived FGF23, which acts on the kidney to suppress phosphate reabsorption and down-regulate CYP27B1 [96–98]. Therefore, FGF23 acts as the primary initiating signal in CKD-MBD pathogenesis. As this cascade progresses, the secondary phase is characterized by a profound drop in active vitamin D synthesis, leading to severely impaired intestinal calcium absorption. This resulting hypocalcemia, combined with ongoing phosphate retention, triggers the tertiary phase: severe secondary hyperparathyroidism. Chronically high PTH fundamentally shifts bone turnover, driving relentless osteoclast activation and culminating in osteitis fibrosa [6,99,100]. Clinical evidence robustly supports this hierarchy, demonstrating that while early FGF23 excess initiates mineral dysregulation, it is the downstream, late-stage collapse of vitamin D and uncontrolled PTH that ultimately results in severe structural skeletal fragility [101,102]. Clinical studies confirm that targeted interventions, such as vitamin D supplementation, can help restore balance when applied appropriately within this temporal cascade [103].

Classic endocrine failures also precipitate OP. Diabetes (T1DM and T2DM) harms bone quality and increases fracture risk via advanced glycation end products that damage collagen structure and reduce osteoblast activity [104,105]. Hyperthyroidism speeds up bone loss [106], and excess glucocorticoids (endogenous or pharmacological) rapidly deplete trabecular bone by disrupting macrophage-fueled osteogenesis [107,108]. In this endocrine–bone axis, estrogen therapy eases bone loss after menopause, which prevents bone loss, reduces fracture risk, and keeps bone structure [109,110]. For patients with hormone deficiencies, therapies such as teriparatide stimulate bone formation [111], while denosumab prevents bone breakdown, including in cancer patients receiving hormone-blocking treatments [112].

Beyond classic endocrine hubs, gastrointestinal malabsorption issues, such as in celiac disease or inflammatory bowel disease, deprive the skeleton of crucial nutrients, severely lowering BMD and raising fracture risk [113]. Moreover, fecal microbiota transplantation prevents bone loss in ovariectomized mice by reshaping gut microbiota and metabolism [114]. Conversely, dysbiosis in metabolic disorders actively destroys bone. Recent findings highlight that obese gut microbiota-derived lipopolysaccharides activate Toll-like receptor 4 signaling to induce bone marrow macrophage senescence. These senescent macrophages hypersecrete grancalcin, driving obesity-associated skeletal deterioration—a pathogenic loop that can be effectively mitigated by grancalcin neutralization [115]. Finally, human studies confirm that muscle mass predicts bone health. From a mechanical standpoint, muscle wasting (sarcopenia) deprives bone of essential mechanical strain and osteogenic myokines, markedly raising fracture risk in older adults [116–118].

### OA and RA

**Fig. 3.**
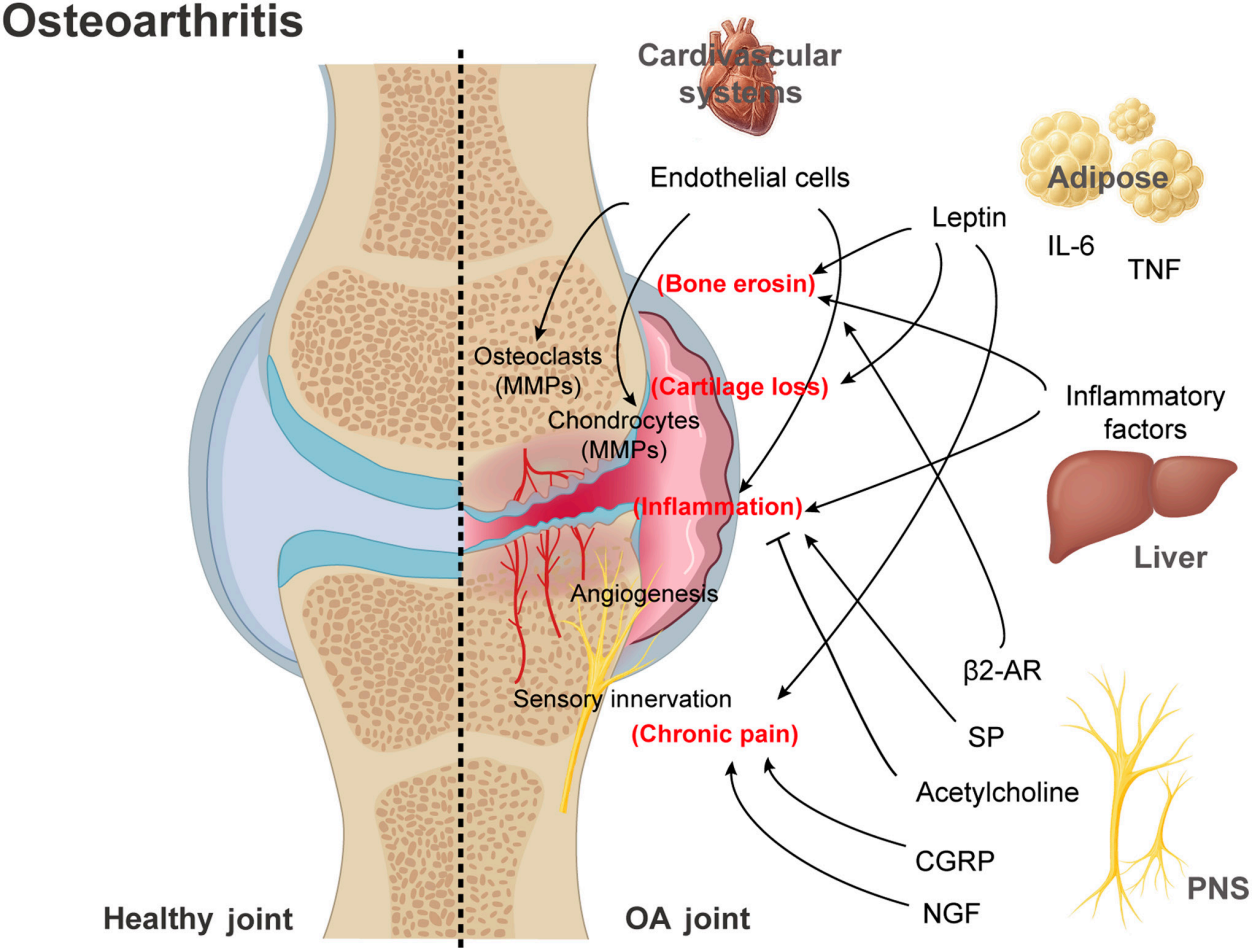
Organ–bone axes in regulating osteoarthritis (OA) pathogenesis. Cardiovascular system, adipose tissue, peripheral nervous system (PNS), and liver are largely involved in OA pathogenesis, including cartilage loss, inflammation, extracellular matrix (ECM) degradation, angiogenesis, and chronic pain.

**Fig. 4.**
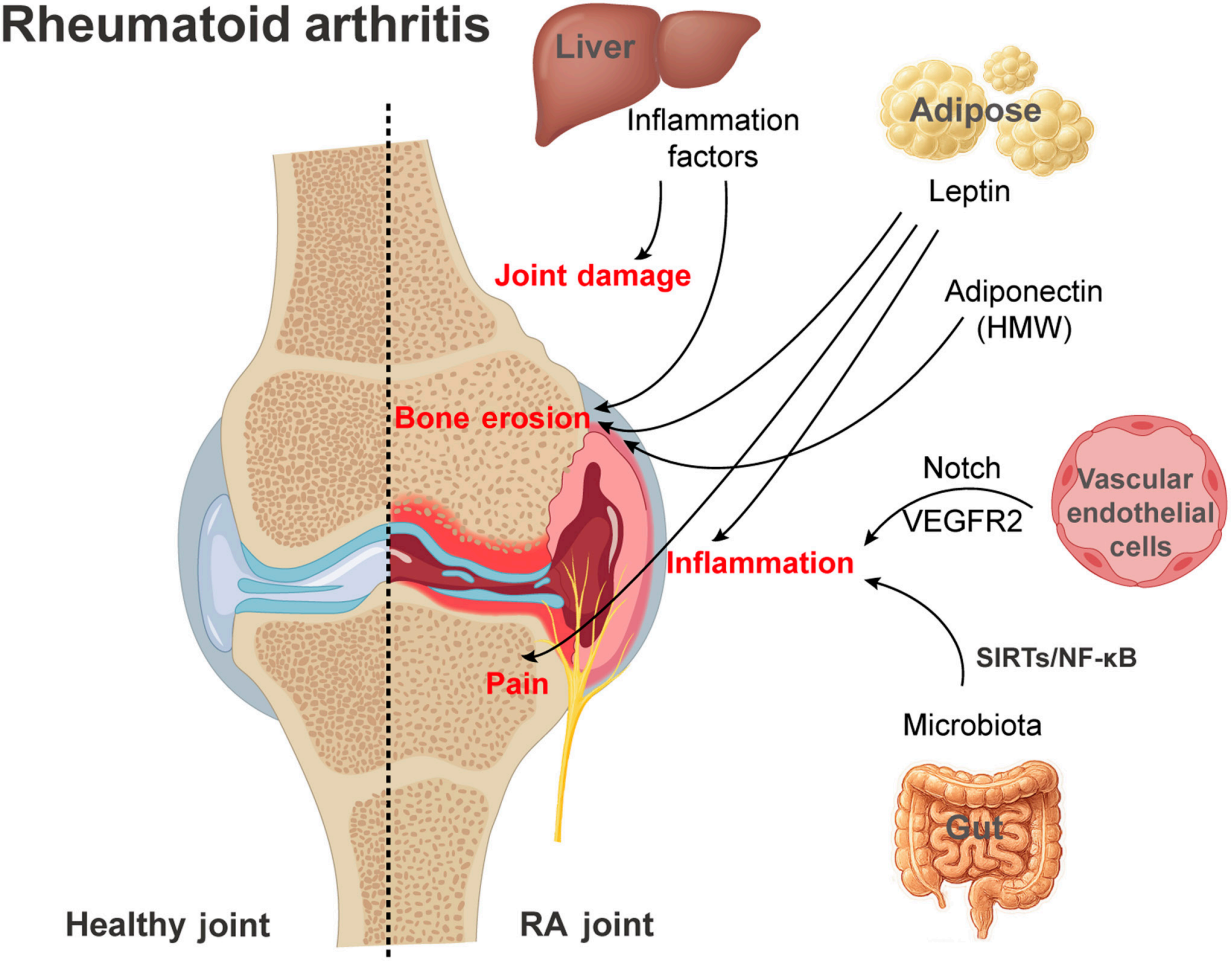
Organ–bone axes in regulating rheumatoid arthritis (RA) pathogenesis. Liver, adipose tissue, vascular system, and gut contribute to the pathology of RA, including bone erosion, inflammation, and pain. VEGFR2, vascular endothelial growth factor receptor 2. HMW, high-molecular-weight.

OA and RA are characterized by severe joint destruction driven by a complex interplay of systemic inflammation, metabolic shifts, and neurovascular dysfunction (Figs. 3 and 4).

Nonalcoholic fatty liver disease and chronic liver disease are associated with osteoarticular disorders, probably through systemic inflammatory and metabolic pathways [119]. In parallel, vascular dysfunction represents a key convergent mechanism: Systemic inflammation promotes endothelial dysfunction, whereas local hypoxia within inflamed joints drives pathological angiogenesis, thereby sustaining synovitis and structural damage [120,121]. Preclinical studies further suggest that vascular endothelial growth factor/vascular endothelial growth factor receptor 2-targeted or endothelial-directed interventions can attenuate synovial angiogenesis and joint inflammation [122,123]. Adipose tissue further amplifies this inflammatory–metabolic network: Leptin derived from obesity or periarticular fat depots promotes synovial inflammation, cartilage catabolism, and pain, whereas adiponectin exerts context-dependent effects, including associations with radiographic progression and bone erosion in RA [124–126].

Neuroimmune dysregulation provides an additional mechanistic layer. In OA, osteoclast-derived netrin-1 and NGF-dependent nociceptive pathways promote aberrant sensory innervation and pain sensitization [127,128]. Conversely, vagal cholinergic activation suppresses proinflammatory cytokine production and alleviates arthritis-like phenotypes in preclinical models [129]. Finally, the gut–joint axis links microbial metabolites to joint inflammation and degeneration. Gut dysbiosis has been implicated in RA-associated immune activation, whereas modulation of microbial BA metabolism and glucagon-like peptide-1 secretion has emerged as a potential therapeutic route in OA [130]. Distinct OA clinical subtypes also exhibit specific microbial-metabolic signatures, exemplified by altered tryptophan metabolism in erosive hand osteoarthritis [131].

### IVDD

**Fig. 5.**
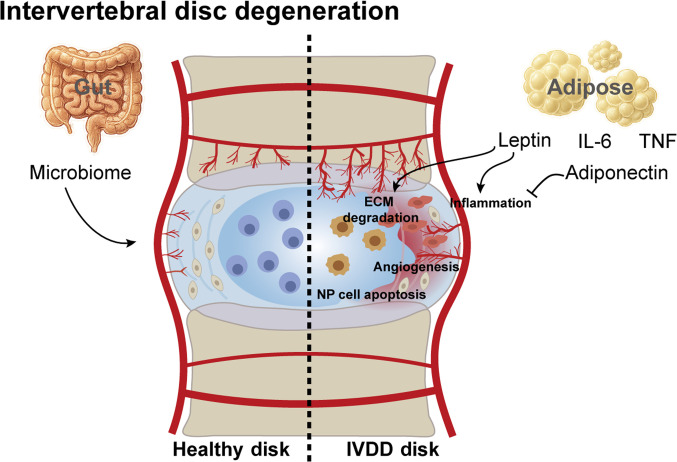
Organ–bone axes in regulating intervertebral disc degeneration (IVDD) pathogenesis. Adipose tissue and gut are reported with essential regulatory functions in IVDD pathogenesis, including extracellular matrix (ECM) degradation, nucleus pulposus (NP) cell apoptosis, and inflammation.

The pathophysiology of IVDD is increasingly recognized as a multiorgan metabolic issue rather than isolated mechanical wear (Fig. 5). Evidence showed that adipose-driven inflammation is a major contributor, and obesity and metabolic traits strongly associate with disc pathology and back pain [132]. Specifically, leptin promotes matrix degradation and inflammation within the disc [133], whereas adiponectin may paradoxically offer protection by lowering proinflammatory cytokines in degenerated nucleus pulposus cells [134]. Beyond metabolic factors, the nervous system is directly implicated in IVDD-associated pain. Both the annulus fibrosus and nucleus pulposus cells express neurotrophins (NGF and brain-derived neurotrophic factor), which enhance pathological innervation and hyperalgesia within the degenerating disc [135]. Amplifying this local pain network, chronic PGE2 elevation—such as in porous vertebral endplates—enhances sensory innervation and drives pain. Notably, low-dose cyclooxygenase-2 inhibition lowers PGE2 to restore proper brain–bone interoception, successfully reducing endplate porosity and spinal pain without impairing bone remodeling [136]. Furthermore, emerging evidence links microbiome shifts to IVDD, suggesting that modifying gut microbiota balance might offer novel systemic avenues to treat spinal degeneration [137].

## Clinical and Research Implications

Recent advances in multiorgan biology have opened new opportunities to diagnose, monitor, and therapeutically modulate organ–bone interactions. Circulating biomarkers and next-generation imaging now allow real-time assessment of how liver, kidney, gut, adipose tissue, muscle, and immune dysregulation influence skeletal health. At the same time, integrative strategies combining systemic biomarkers, organ-directed therapies, lifestyle interventions, and computational prediction are shifting clinical practice from isolated bone-centered management toward a multiorgan precision framework, improving risk stratification and therapeutic decision-making.

### Translational and therapeutics insights

Based on the bidirectional communications between bone and other organs, treatments that target other organs can help to promote bone health, which may address root causes linked to bone diseases (Table 2). For example, several liver-related drugs and treatments are reported with beneficial effects in bone conditions. Bisphosphonates such as alendronate reduce bone loss and lower fracture rates after transplantation [138]. Statins were initially used to lower blood lipids; research confirms that statins increase BMP-2 expression in bone cells, leading to better bone formation. Moreover, vitamin D deficiency is nearly universal in liver disease, and switching to bone-safer antivirals such as tenofovir alafenamide improves spinal bone density in chronic hepatitis B [139].

**Table 2.** Therapeutic strategies acting on other organs to improve bone health

Target Organ/System	Intervention and representative agents	Bone-protective mechanism	Key evidence (Ref.)
Liver–bone axis	• Bisphosphonates (e.g., alendronate)	Reduce posttransplant bone loss and fracture risk	[138]
	• Sirtuin activators (“liver support”)	Lower hepatic lipid toxicity, enhance osteoblast activity	[177]
	• Sirtuins in bone cells	Promote bone growth and remodeling balance	[178]
	• Vitamin D and calcium supplementation	Offset malabsorption, improve mineralization	[179]
	• Antiviral therapy for hepatitis	Restores liver function and improves BMD	[139]
	• Statins	Increase BMP-2 expression, stimulate osteogenesis	[180]
Gut–bone axis	• Probiotics (e.g., *Lactobacillus* spp.)	Boost SCFA production, reduce inflammation, enhance calcium absorption	[55]
	• Prebiotic fibers	Support microbiota composition and calcium uptake	[181]
	• Fecal microbiota transplantation (FMT)	Reshape gut microbiota, prevent OVX-induced bone loss, potential benefit in RA	[114]
Endocrine–bone axis	• Estrogen replacement	Prevent postmenopausal bone loss, reduce fractures	[109,110]
	• Teriparatide (PTH analog)	Stimulate bone formation in severe osteoporosis	[111]
	• Denosumab (RANKL inhibitor)	Block osteoclast-mediated bone resorption, including in cancer therapy	[182]
Immune modulation	• Anti-TNF agents	Decrease inflammation and bone erosion in arthritis	[183]
Neural negulation	• β-blockers	Counteract sympathetic overactivity and stress-related bone loss	[184]
Muscle–bone crosstalk	• Myostatin inhibitors	Increase muscle mass and indirectly enhance bone strength	[185]
Lifestyle and nutritional/pharmacological strategies	• Resistance/weight-bearing exercise	Stimulate mechanotransduction, improve BMD	[186]
	• Aerobic exercise + vitamin D and calcium	Reduce fracture risk, slow age-related bone loss	[50,140]
	• Aerobic training + ω-3 fatty acids	Lower inflammation, slow postmenopausal bone loss	[141]
	• Exercise + bisphosphonate therapy	Greater BMD gain than drug alone	[187]
	• GLP-1 agonists + exercise during weight loss	Preserve bone mass	[142]
	• Adequate dietary protein	Support collagen matrix and bone mineralization	[188]
	• Magnesium intake	Associated with higher BMD in men and women	[189]
	• Kidney disease management	Restore Ca^2+^ and mineral balance, reduce fracture risk	[144]
Precision and regenerative medicine	• Genetic screening of Wnt pathway (LRP5/6 variants)	Identify candidates for sclerostin inhibition (e.g., romosozumab)	[150,169]
	• Bone organoids / engineered tissue	Preclinical drug testing and patient-specific grafting	[145]
	• Organ-on-a-chip	Simulate individual drug responses, reduce side effects	[146]
	• Multiomics integration (genomics, proteomics, metabolomics)	Reveal gut–bone metabolic links, guide interventions	[147,190]
	• Artificial intelligence	Integrate datasets to predict risk and tailor therapy	[148]

SCFA, short-chain fatty acid; TNF, tumor necrosis factor; BMD, bone mineral density; GLP-1, glucagon-like peptide-1, PTH, parathyroid hormone; OVX, ovariectomy; LRP5/6, low-density lipoprotein receptor-related protein 5/6; RANKL, receptor activator of nuclear factor κB ligand

Besides using drugs or treatments alone, combining lifestyle and pharmacological strategies provides the greatest benefit for bone health (Table 2). Regular resistance and weight-bearing exercise builds muscle and supports bone formation, while aerobic activity paired with adequate calcium intake and vitamin D lowers fracture risk and slows age-related bone loss [140]. Long-term aerobic training with omega-3 fatty acids helps reduce inflammation and has been shown to slow postmenopausal bone loss [141]. In patients losing weight, adding glucagon-like peptide-1 therapy to an exercise program helps preserve bone mass [142]. Lifestyle measures that protect cardiovascular and kidney health—including control of blood pressure and blood glucose—also benefit the skeleton, since diabetes and kidney disease impair bone through mineral dysregulation and oxidative stress [143,144]. Together, these approaches highlight that integrating targeted medications with structured exercise and optimal nutrition offers a stronger defense against OP and related skeletal disorders than any single intervention.

Bone organoids and other organ models now simulate human bone structure and function, allowing researchers to test drugs, explore organ interactions, and plan individualized therapies before clinical use [145]. Organ-on-a-chip can simulate personalized drug responses, reduce side effects, and improve efficacy [146]. Multiomics analyses—integrating gut metagenomic and metabolomic data—reveal connections between the microbiota and bone metabolism, identifying amino acid metabolism pathways and specific microbial taxa as potential targets for intervention [147]. Artificial intelligence integrates these datasets to identify high-risk patients and recommend tailored prevention or treatment strategies [148]. Genetic characterization of Wnt pathway components—particularly the osteocyte-derived Wnt inhibitor sclerostin—demonstrates that molecular and genotype-based stratification can predict individual therapeutic responses and guide precision selection of bone-anabolic interventions [149,150]. Together, these advances show how genetic screening, organ modeling, and engineered bone tissue can integrate organ signals with skeletal biology to improve outcomes.

In addition to circulating factors, imaging advances reveal organ-specific impacts in physical and pathological conditions (Table 3). Dual-energy x-ray absorptiometry (DXA) measures areal BMD but lacks sensitivity for microstructural deterioration. High-resolution peripheral quantitative computed tomography assesses cortical and trabecular architecture, linking kidney dysfunction to porosity [151]. Magnetic resonance imaging detects marrow fat infiltration from adipose dysregulation [152]. Beyond structural imaging, functional and emerging modalities further support organ–bone assessment. Positron emission tomography/computed tomography quantifies synovial inflammatory burden in RA [153]. Opportunistic computed tomography identifies vertebral fractures and estimates BMD from routine clinical scans, enabling early detection of OP without additional radiation burden [154]; quantitative ultrasound predicts fracture risk independently of DXA, and musculoskeletal ultrasound assesses sarcopenia [155,156]. Artificial intelligence-integrated platforms combining DXA, high-resolution peripheral quantitative computed tomography, and magnetic resonance imaging enable holistic risk prediction across organ axes, advancing precision diagnosis of skeletal diseases [148].

**Table 3.** Imaging advances in organ–bone crosstalk

Modality	Key applications	Organ-specific insights	Limitations/Advances	References
Dual-energy x-ray absorptiometry (DXA)	Measures areal BMD at radius/spine/hip; standard for osteoporosis diagnosis.	Detects gross density loss from endocrine/adipose dysregulation.	Misses microstructure; supplemented by fracture risk tools.	[191]
High-resolution peripheral quantitative computed tomography (HR-pQCT)	Assesses volumetric BMD, cortical/trabecular architecture at radius/tibia.	Links kidney dysfunction to cortical porosity; evaluates muscle–bone interfaces.	Peripheral sites only; AI integration for porosity modeling.	[151,192]
Magnetic resonance imaging (MRI)	Visualizes bone marrow composition; quantifies fat infiltration.	Reveals adipose-driven marrow adiposity in obesity/diabetes.	High cost; emerging protocols for quantitative fat mapping.	[152]
Positron emission tomography/computed tomography (PET/CT)	Detects synovial metabolic activity and quantifies inflammatory burden in RA joints.	Highlights immune-mediated pathology.	Radiation exposure; useful for staging inflammatory diseases.	[153]
Opportunistic CT	Detects vertebral fractures; estimates BMD from routine CT scans.	Supports early detection of osteoporosis-related vertebral fragility from incidental CT imaging.	Radiation exposure; BMD calibration not standardized	[154]
Ultrasound (QUS / MSUS)	QUS predicts fracture risk independently of DXA; MSUS quantifies muscle thickness and echo intensity for sarcopenia.	Noninvasive assessment of sarcopenia-osteoporosis overlap.	Operator-dependent; QUS heel site differs from DXA skeletal sites	[155,156]
AI-integrated multimodal imaging	Predictive modeling from combined DXA/HR-pQCT/MRI data.	Holistic risk prediction across organ axes; refines diagnosis in complex cases.	Data integration challenges; improves precision medicine.	[148]

RA, rheumatoid arthritis; BMD, bone mineral density; AI, artificial intelligence; QUS, quantitative ultrasound; MSUS, musculoskeletal ultrasound

### Current progress and future directions

The growing body of literature highlights how organs influence skeletal health. However, despite major progress, critical knowledge gaps limit the translation of these findings into clinical practice and slow the development of effective, organ-targeted therapies for skeletal diseases. Key challenges fall into 3 areas: defining mechanistic underpinnings with sufficient cellular and molecular resolution; stratifying disease heterogeneity into biologically meaningful subtypes; and addressing longitudinal data deficiencies to capture the evolution of multiorgan networks. To provide a clearer translational overview, we have summarized representative organ–bone-axis–based interventions across OP, OA, and RA (Table 4). For each modality, we indicate the clinical stage—from preclinical studies to approved therapies—and highlight key translational challenges.

**Table 4.** Organ–bone-targeted therapies: Current clinical stage and limitations

Diseases	Therapeutic target / axis	Sample size	Current clinical stage	Major challenges / limitations	Clinical trial
Osteopenia / early postmenopausal bone loss	Gut–bone axis (oral *Lactobacillus* probiotic supplementation)	249	Randomized, double-blind, placebo-controlled clinical trial (phase N/A)	Modest effect size; heterogeneous microbiome response; mechanism unclear; limited long-term data.	NCT02722980 [193]
Postmenopausal bone loss / osteopenia	Gut–bone axis (oral *Lactobacillus plantarum* + *L. paracasei*)	170	Phase not specified; randomized, double-blind, placebo-controlled trial; no published results	Outcome limited to BMD; probiotic strain variability; unclear durability	NCT06375668
Age-related bone loss	Gut–bone axis (oral synbiotic SBD111: probiotic + prebiotic)	220	Randomized, double-blind, placebo-controlled clinical food trial(ongoing; no published results)	Long duration (18 months); medical food category—not drug; no results yet; BMD end point only	NCT06389539
Metabolic knee OA + obesity	Gut–joint axis (oral prebiotic: oligofructose-enriched inulin)	54	Randomized, placebo-controlled clinical trial; no published results	No results; long duration; OA pain subjective variability; mechanistic end points exploratory	NCT04172688
OA	Gut–joint axis(oral probiotic)	40	Interventional (phase N/A); no published results	Very small sample; unclear completion; no efficacy data; exploratory only	NCT03985709
OA (pain-predominant)	Gut–joint axis(oral probiotic)	50	Randomized controlled trial; no published results	Unclear completion; no efficacy data; exploratory	NCT03968770
RA (MTX-refractory)	Gut–joint axis (fecal microbiota transplantation)	30	Phase 2 randomized, double-blind, parallel-group trial	Small sample; single center; donor variability; uncertain long-term efficacy/safety	NCT03944096
RA (refractory)	Neuro–immune axis (active implantable vagus nerve stimulation device	18	Randomized neuromodulation device trial (phase N/A)	Small cohort; invasive; limited long-term data	NCT01552941 [194]
RA (multidrug-refractory)	Neuro–immune axis (miniaturized vagus nerve stimulation device)	14	Two-stage pilot study (randomized, sham-controlled)	Small cohort; invasive; limited power for efficacy; limited long-term data	NCT03437473 [195]
RA (csDMARD-inadequate responders; biologic-naïve)	Neuro–immune axis (auricular vagus nerve stimulation)	113	Randomized, double-blind, sham-controlled clinical trial	No efficacy; short follow-up; limited stimulation strength	NCT04306744 [196]
RA (biologic/tsDMARD-inadequate responders)	Neuro–immune axis (implantable cervical vagus nerve stimulation)	60 (Stage 1)	Randomized, double-blind, sham-controlled pivotal trial	Implant required; perioperative risks (hoarseness/vocal cord paresis); short follow-up; efficacy not assessed in Stage 1	NCT04539964 [197]
RA (biologic/tsDMARD-inadequate responders)	Neuro–immune axis (vagus nerve-targeted neuromodulation system)	242 (full pivotal trial)	Pivotal randomized, double-blind, sham-controlled trial	Implant required; modest benefit; perioperative risks; limited long-term data	NCT04539964 [198]

OA, osteoarthritis; RA, rheumatoid arthritis; BMD, bone mineral density; csDMARD, conventional synthetic disease-modifying antirheumatic drug; tsDMARD, targeted synthetic disease-modifying antirheumatic drug; N/A, not applicable; MTX, methotrexate

#### Mechanistic underpinnings

Although several signaling axes have been identified—for example, FGF23 from the kidney, leptin from adipose tissue, and sympathetic innervation from the nervous system—the full spectrum of molecular mediators is far from clear. Recent studies have shown that osteokines participate in a 2-way dialogue between bone and other organs, but causal relationships are less well defined [157,158]. The roles of extracellular vesicles, microRNAs, and metabolic intermediates remain underexplored. For instance, the liver secretome includes a wide range of cytokines and metabolic regulators, but only a handful have been studied in relation to bone remodeling. Recent advances in single-cell RNA sequencing, spatial transcriptomics, and proteomics show that organ–bone communication is often mediated by discrete cell subsets [159,160]. However, most studies are still limited to the analysis of single organ or tissue, and spatial transcriptomic, metabolomic, or proteomic analyses that explicitly interrogate how remote organs remodel the bone microenvironment are still rare. A priority for the next decade is to define these specialized subpopulations across organs and to map, at single-cell resolution, the ligand–receptor and vesicle-mediated circuits by which they influence bone cells. Most studies consider each organ in isolation, but bone health is shaped by the integration of multiple organ inputs. For example, CKD alters vitamin D metabolism (renal effect), induces systemic inflammation (immune effect), and disrupts cardiovascular homeostasis, all of which converge on bone fragility. How these organ-derived inputs interact remains poorly understood. One testable hypothesis is that specific combinations of organ stressors (for example, hepatic lipotoxicity plus gut dysbiosis, or CKD plus heightened sympathetic tone) generate emergent transcriptional states in osteoclast and osteoblast precursors that cannot be predicted from single-organ perturbations alone. Another is that neural inputs from sympathetic and sensory fibers regulate bone in a circuit-specific and spatially restricted manner, rather than uniformly across the skeleton. Although evidence supports roles for sympathetic and sensory nerves in bone metabolism, the precise mechanisms remain debated. Some studies show that sympathetic activation promotes bone loss, while others suggest context-dependent effects. Similarly, parasympathetic influences and neuropeptides such as SP, CGRP, and neuropeptide Y have been linked to bone remodeling, but their integration with immune and endocrine pathways is unclear [12,161]. Addressing these questions will require cross-organ perturbation models combined with computational approaches that construct dynamic organ–bone network models integrating multiomics and spatial information. A deeper understanding of neural circuits, neuroimmune crosstalk, and neurotrophic factor signaling in skeletal biology is required, which may ultimately inform therapeutic strategies targeting the emerging brain–bone axis [162].

#### Disease heterogeneity

Bone diseases exhibit marked heterogeneity in clinical presentation and pathology but share partially overlapping mechanisms. OP and arthritis (OA and RA) are distinct entities, yet all involve the skeleton and multiple organ systems. Nevertheless, common threads exist: Obesity increases the risk of both OP and OA; liver dysfunction can aggravate RA; renal impairment accelerates bone loss and mineral disorders in all 3 conditions. Epidemiological data indicate that RA patients have approximately double the prevalence of OP compared with the general population, and RA is associated with higher fracture risk driven by chronic inflammation, in contrast to OA where localized cartilage damage and subchondral bone changes dominate [163]. However, cross-disease analyses integrating shared organ–bone signatures remain limited.

An important conceptual advance will be to move from purely phenotype-based diagnoses toward organ-axis–based subtypes that can guide individualized treatment strategies. For OP, for example, one can envisage “liver-dominant”, “gut-immune”, and “neurometabolic” subtypes, defined by characteristic patterns of hepatokines and IGF-1 [23]. microbiome-derived metabolites and Th17 polarization [55,60], respectively. Patients with liver-dominant signatures may preferentially benefit from anabolic agents combined with optimization of hepatic function [164]; those with gut-immune signatures may be candidates for targeted microbiome modulation or IL-17/IL-22 pathway blockade [114]; individuals with neurometabolic signatures might respond better to disease-specific neural interventions: β-Adrenergic blockade could serve as an effective adjunct to standard OP therapies [165], whereas targeted neuromodulation (e.g., vagus nerve stimulation) holds distinct promise for alleviating inflammation and pain in osteoarthritis [129,166]. A central hypothesis is that such organ-axis molecular signatures will predict fracture risk and therapeutic response more accurately than bone density or joint imaging alone. Preclinical models and early-phase trials should therefore be designed to enrich for these subtypes, rather than treating OP or arthritis as monolithic entities.

In addition to patient heterogeneity, experimental models require refinement. Current animal models of bone disease often rely on ovariectomy, glucocorticoids, or inflammatory stimuli to mimic OP or arthritis, but they capture only limited aspects of human pathology and typically perturb a single organ axis at a time. Most OA, OP, and RA animal models rarely integrate concomitant gut dysbiosis, adipose inflammation, or neural dysregulation. These limitations likely contribute to the high failure rate of drug candidates that perform well in animal studies but fail in clinical trials [167]. Given that bone research still relies heavily on animal models for mechanistic insight, careful selection and, where possible, combination of models that incorporate multiorgan perturbations will be crucial, particularly for OA drug development [168].

#### Longitudinal data deficiency

Most current studies provide cross-sectional snapshots of organ–bone interactions without longitudinal follow-up. This obscures the temporal sequence by which organ dysfunction precedes, coincides with, or follows skeletal deterioration. For liver disease, we do not know if bone loss hits early or late. In kidney disease, when do mineral changes cause weak bones? Gut changes during menopause and bone effects lack timelines too. Long-term longitudinal studies with repeated multiorgan assessments are therefore required. Bone-derived signals exert systemic effects across spatial and temporal scales, but we still lack the data of long-term investigation. For example, bone-derived factors affect metabolism and blood flow with long-term effects [158]. Thus, we need standard long-term groups to find causes, markers, and patient types.

A key future direction is therefore to develop dynamic, multiorgan cohorts that integrate imaging, function tests, and multiomics data across tissues. One testable hypothesis is that defined temporal patterns of organ dysfunction, such as liver inflammation, adipose insulin resistance, and sympathetic activation, generate recognizable trajectories of bone loss that can be predicted from an integrated organ–bone network model. Another is that modulating a single organ axis (for example, the gut microbiome) in stratified patients will propagate measurable changes through the network, normalizing both organ function and skeletal end points. Designing clinical trials that target specific organ axes stratify patients by organ-dominant subtype and incorporate repeated multiorgan measurements will be essential to validate these models and to translate interorgan communication biology into genuinely individualized skeletal therapies.

## Conclusion

Recent studies underscore the pivotal role of interorgan communication in bone homeostasis. Organs such as muscle, liver, brain, and adipose tissue influence skeletal health through secreted factors, extracellular vesicles, and signaling pathways. Bone diseases are systemic problems. Despite advances in the understanding of organ–bone interactions, key gaps remain. Mechanistic studies need finer resolution at the cellular and molecular levels, including neural and cross-organ communications. Disease heterogeneity highlights the importance of patient stratification and standardized models. Finally, longitudinal and multiomics approaches are needed to capture the dynamic nature of organ–bone communication. Addressing these challenges will require collaboration across endocrinology, rheumatology, immunology, metabolism, neuroscience, and computational biology. Only through such integration can the field move from descriptive associations toward mechanistic understanding and effective therapies.

## Data Availability

Data availability is not applicable to this article as no new data were created or analyzed in this study.
